# The impact of information presentation on self-other risk decision-making

**DOI:** 10.3389/fpsyg.2024.1357644

**Published:** 2024-05-09

**Authors:** Ai-Bao Zhou, Ze-Kai Li, Pei Xie, Yun-Fei Lei, Bai-Xia Cui, Le Yao, Chao-Zheng Huang

**Affiliations:** ^1^School of Psychology, Northwest Normal University, Lanzhou, China; ^2^School of Judicial Police, Gansu University of Political Science and Law, Lanzhou, China; ^3^School of Psychology, Sichuan Normal University, Chengdu, China

**Keywords:** risk decision making, social distance, decision from description, decision from experience, self-other

## Abstract

To explore the impact of social distance and information presentation types on self-other risk preferences in monetary tasks. Risk preferences were examined in decision-making tasks and experiential information tasks within different frameworks when participants made decisions for themselves and others. Experiment 1 employed experiential decision tasks and revealed individual differences in decision-making for oneself and others. In gain situations, participants exhibited more risk aversion when deciding for others compared to themselves. Experiment 2 presented both types of information simultaneously to investigate whether risk decisions for oneself and others are influenced by information types. Results indicated that experiential information led participants to make more conservative choices for others, while descriptive information eliminated this effect. This study discovered the influence of social distance on self-other risk decisions and the role of information presentation types in self and other risk decision-making. Future research could further explore self-other decision-making from the perspectives of decision-makers’ traits and culture.

## 1 Introduction

In social life, decisions of various scales constitute the everyday lives of individuals. People not only make decisions for themselves, but sometimes they also need to provide advice or even make decisions on behalf of others. When making decisions, multiple factors are typically considered to determine the best course of action, especially when outcomes involve significant uncertainty and various possibilities (i.e., risk decisions). The prediction of events is also influenced by decision-related information. Yates (1992) defined risk decision-making as the process of making optimal choices in situations characterized by uncertainty and disparities in potential gains and losses. When the object of risk decision-making changes, people’s decision-making also varies accordingly. Early research has found inconsistencies in individuals’ performance when making decisions for themselves compared to making decisions for others (Hsee and Weberer, 1997), particularly in terms of differential risk tendencies between these two types of decisions. Researchers have referred to this disparity as the “self-other difference in decision making” (Liu et al., 2018). The emergence of self-other decision-making differences is most directly linked to the psychological distance between the decision-maker and the decision subject (Polman, 2012; Liu et al., 2014). Some studies in contexts involving financial and interpersonal risk decisions have found that individuals exhibit a higher risk tendency when making decisions for others as opposed to themselves (Stone and Allgaier, 2008; Batteux et al., 2017). However, other research in the same decision-making contexts has discovered that individuals tend to be less risk-averse when making decisions on behalf of others than when making decisions for themselves (Eriksen and Kvaløy, 2010; Petrova et al., 2016). Although these studies were conducted in the context of financial and interpersonal risk decision-making, the specific decision-making frameworks in the studies may have influenced participants’ risk preferences. Differences in the way risk is presented (e.g., loss versus gain frameworks) may lead to different perceptions of risk when making decisions for others.

According to the Construal Level Theory (CLT), psychological distance can significantly impact an individual’s cognition and behavior. The farther an object or person is perceived to be from our psychological proximity, the less detailed and subjective our understanding of them becomes (Trope et al., 2007). Psychological distance, which encompasses temporal distance, spatial distance, probability distance, and social distance, refers to the perceived cognitive separation between an individual and objects or individuals in their environment (Liberman and Trope, 1998). Social distance, in particular, is considered the subjective experience of intimacy between oneself and others (Zhang et al., 2019). While early research has explored the general performance of individuals when making risk decisions on behalf of others, the Risk-as-Feelings theory proposed by Loewenstein et al. (2001) suggests that decision-making disparities arise due to the differential emotional outcomes anticipated by individuals when deciding for themselves versus for others. Specifically, when making decisions for others, individuals’ perception of risk is lower compared to making decisions for themselves (Loewenstein et al., 2001). This discrepancy is attributed to the variance in emotional responses anticipated from potential outcomes, whether deciding for oneself or on behalf of another. Other studies have found that within the context of risk decision-making, decision-makers, when making choices for others, tend to exhibit risk aversion in loss scenarios and risk-seeking behavior in gain situations, compared to their decisions for themselves (Zhang et al., 2017). Their findings demonstrate that decisions made for others are influenced by the social distance between the decision-maker and the recipient, which in turn affects risk aversion and risk-seeking behaviors. Recent research, for instance, has manipulated social distance to investigate individuals’ risk preferences when making decisions for themselves and for others. Results revealed that as social distance increased, people tended to lean towards neutral-risk choices (Sun et al., 2020). Studies that have not explicitly defined psychological distance between oneself and others in risk decision tasks have often found similarities in risk preferences when deciding for oneself and for others (Benjamin and Robbins, 2007). These findings suggest that discrepancies in results may arise from a lack of clear delineation regarding the identity of the decision recipient in the studies. Therefore, this study manipulates social distance between the decision-maker and the surrogate decision-maker within both gain and loss frames to explore the characteristics of risk decision-making for others with varying degrees of social distance. According to extant theories, individuals tend to favor options with lower risk when making decisions for themselves. However, when making decisions on behalf of others who are greater social distance, subjects may opt for options that entail higher risk.

Additionally, certain factors can modulate the outcomes of self-other risk decision-making, such as self-depletion and value orientations (Yang et al., 2018; Ren et al., 2021). Early research in the field of risk decision-making predominantly involved the presentation of descriptive information, which entailed explicitly providing subjects with the probability outcomes of each option (Thaler, 1980). Through this mode of presenting decision content, researchers inferred general patterns in decision-making, including phenomena such as the endowment effect (Thaler, 1980), prospect theory (Kahneman and Tversky, 1979; Tversky and Kahneman, 1992), and the asymmetric dominance effect (Huber et al., 1982). However, an increasing number of researchers have observed that real-life risk decisions often lack detailed probabilistic information and frequently necessitate decisions based on personal experiences. Hertwig et al. (2004) distinguish descriptive decision-making as a method based on known probabilities for all options, whereas experiential decision-making relies on the decision-maker’s personal experiences. Earlier studies indicated that when subjects make decisions based on experience, approximately 66% of individuals tend to make decisions that lean towards higher risk (Barron and Erev, 2003). These discrepancies in the content of decision information lead to divergent outcomes, particularly in terms of their impact on risk preferences (Hertwig and Erev, 2009).

Previous research on self-other decision-making predominantly involved the presentation of descriptive information. Numerous studies have consistently shown that individuals who make decisions on behalf of others exhibit a higher propensity for risk-taking compared to when making decisions for themselves (Chakravarty et al., 2011; Batteux et al., 2017). Yet, it remains to be seen whether different patterns emerge when information is presented experientially. Moreover, in real-life scenarios, individuals encounter risk decision information that may be more complex, often involving the simultaneous presentation of both descriptive and experiential information. Under these influences, individuals’ risk decision behaviors may be substantially affected. According to the antisocial orientation hypothesis proposed by Olschewski et al. (2019), individuals often exhibit heightened risk aversion when making decisions for others, aiming to maximize their own rewards and gain an advantage over others. Another perspective explains the risk aversion in decisions for others when experiential information is used. In such cases, individuals may feel a latent sense of responsibility, which motivates them to opt for conservative options to prevent potential blame in case of an erroneous guess (Leonhardt et al., 2011). However, whether different types of information can alter this conservative decision-making when individuals make risk decisions for psychologically others is a matter that requires further investigation. To investigate the influence of information presentation modes on self-other decision-making in risk decisions, the study employed risk decision tasks within gain and loss frames. Classic social distance manipulation techniques were used in the experiments. Furthermore, based on this foundation, the study delved into the impact of information presentation modes under gain and loss frames on self-other decision-making.

## 2 Experiment 1: the impact of experiential information on self-other risk decision-making

### 2.1 Purpose and hypothesis

Adopting a 2 (Decision Target: Self vs. Other) × 2 (Frame Condition: Gain vs. Loss) mixed-design experiment. The independent variables were Decision Target (between-group variable) and Frame Condition (within-group variable), with the dependent variable being risk preference. Adopting experiential decision tasks, participants were asked to make risk decisions for themselves and others, aiming to explore the impact of experiential information presentation on self and other risk decision-making. Based on existing research, the hypothesis posits that there are differences in risk preferences when making decisions for oneself and others under experiential information conditions.

### 2.2 Methods

#### 2.2.1 Participants

Using G-power 3.1 software, the required total number of participants under the conditions of effect size (Effect size) of 0.25 and statistical power (Power) of 0.8 is 46. Sixty-nine university students were recruited for the current experiment, including 46 females and 23 males, *M_age_* = 22.03, *SD* = 2.63. All participants were right-handed, and their vision or corrected vision was normal. Participants were randomly assigned to the self (*n* = 35) and other (*n* = 34) groups. All participants volunteered and had not participated in similar experiments before. The experiment obtained approval from the Ethics Committee of Northwest Normal University, and all participants provided written informed consent. Before the experiment, participants were informed that they would receive a compensation of 20 yuan upon completion of the experiment.

#### 2.2.2 Materials

The decision scenarios utilized classic monetary decision tasks as the research material. The premise framework for risk decision-making was divided into gain and loss, with each of these frames further categorized into conservative and risky options. Subjects are required to complete six distinct decision-making tasks, detailed in Table 1, where the first three tasks are set in gain contexts, and the last three are in loss contexts. Each decision task presents two options, for example, a risky option: an 80% chance of winning 25 yuan and a 20% chance of winning nothing, versus a conservative option B: a 100% chance of winning 20 yuan. The decision tasks in the loss context correspond to those in the gain context, with the only difference being that the amounts to be won are replaced with amounts to be lost. Within each context, the three tasks are categorized by probability levels: high, medium, and low. These scenarios are described through textual narratives. The decision-making context materials comprise three scenarios each under conditions of gain and loss, with the decision scenarios presented randomly.

**Table 1:** Experiment 1 decision-making tasks.

Frame condition	Tasks	Risky option	Conservative option
Gain	1	80%, 25; 20%; 0	100%, 20
	2	50%, 40; 50%, 0	100%, 20
	3	20%, 100; 80%, 0	100%, 20
Loss	4	80%, −25; 20%; 0	100%, −20
	5	50%, −40; 50%, 0	100%, −20
	6	20%, −100; 80%, 0	100%, −20

#### 2.2.3 Procedure

The experiment took place in a quiet behavioral laboratory. The experimental procedure employed E-prime 2.0 software to present all video stimuli on a 14-inch computer LCD screen with a resolution of 1,600 pixels × 900 pixels. The participants’ eyes were approximately 60 cm away from the screen. Upon entering the laboratory, participants completed basic information forms before commencing the experiment. Instructions on the screen informed participants that they would need to make decisions regarding different scenarios that would appear later. Before to the formal decision-making process, we input the names of others are those of our research assistants (whom the subjects neither know nor are familiar with) into the computer. In the text displayed afterward, these names will appear in specific locations. For subjects within the self-group, the equivalent section of the text they see will be replaced with “you.”

**Figure 1:**
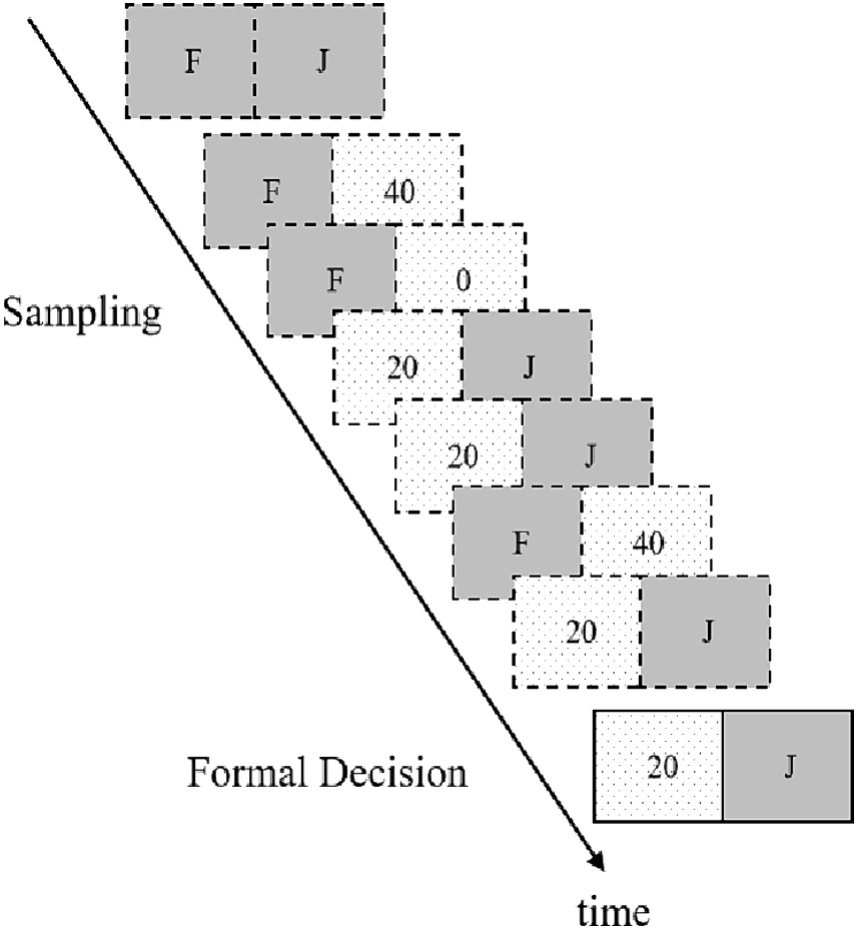
Empirical decision-making paradigm flowchart.

This experiment employs the sampling paradigm from the empirical decision-making framework to investigate the differences between self and other decisions. The experimental procedure is presented using E-Prime 2.0, requiring subjects to complete six decision-making tasks randomly. The specifics of the sampling paradigm are illustrated in Figure 1. On the screen, two buttons labeled “F” and “J” are displayed simultaneously on both sides, with each decision task divided into a sampling phase and an official selection phase. During the sampling phase, subjects are required to press the corresponding button to make sampling choices, which are designed to familiarize them with the outcome information associated with each option. The system provides outcome feedback based on pre-set probabilities for the chosen button (For instance, pressing F always resulted in gaining 20 yuan, while pressing J sometimes resulted in gaining 40 yuan and sometimes 0 yuan), with the result lasting for 2 s. Subjects can press multiple times during this phase to receive feedback, gaining an understanding of the potential outcomes of each button press. There is no limit on the time or number of presses in this phase, so the more a subject samples, the more accurately they will grasp the probabilities and outcome information for each option. To ensure subjects accurately comprehend the probabilities and outcome information associated with each option, they are instructed to sample each option more than 10 times during the sampling phase, closely matching the probabilities associated with each option. This approach aims to minimize the impact of sampling error on the experimental results due to insufficient sampling. After repeated sampling choices, once subjects feel they have adequately understood the probabilities and outcome information for each option, they can proceed to the official selection phase. In the official selection phase, subjects must make a formal decision based on their understanding of the two options, without receiving any feedback for this decision. They were instructed to score 1 point when choosing the risky option and 0 points when choosing the conservative option.

For the self-group, the instructions before the experiment begin are as follows: “*Imagine this is a decision-making scenario you encounter in real life, where choosing different options will lead to different outcomes. How would you choose? Before making a formal decision, you will go through a sampling phase, which allows you to understand the outcomes of each option, until you believe you have sufficiently understood each option before making a choice*.” For the other-group, the instructions are: “*Imagine this is a decision-making scenario encountered by someone you do not know in real life, where choosing different options will lead to different outcomes. Now, you need to make a choice on their behalf. How would you choose? Before making a formal decision, you will go through a sampling phase, which allows you to understand the outcomes of each option, until you believe you have sufficiently understood each option before making a choice*.”

### 2.3 Results

**Figure 2:**
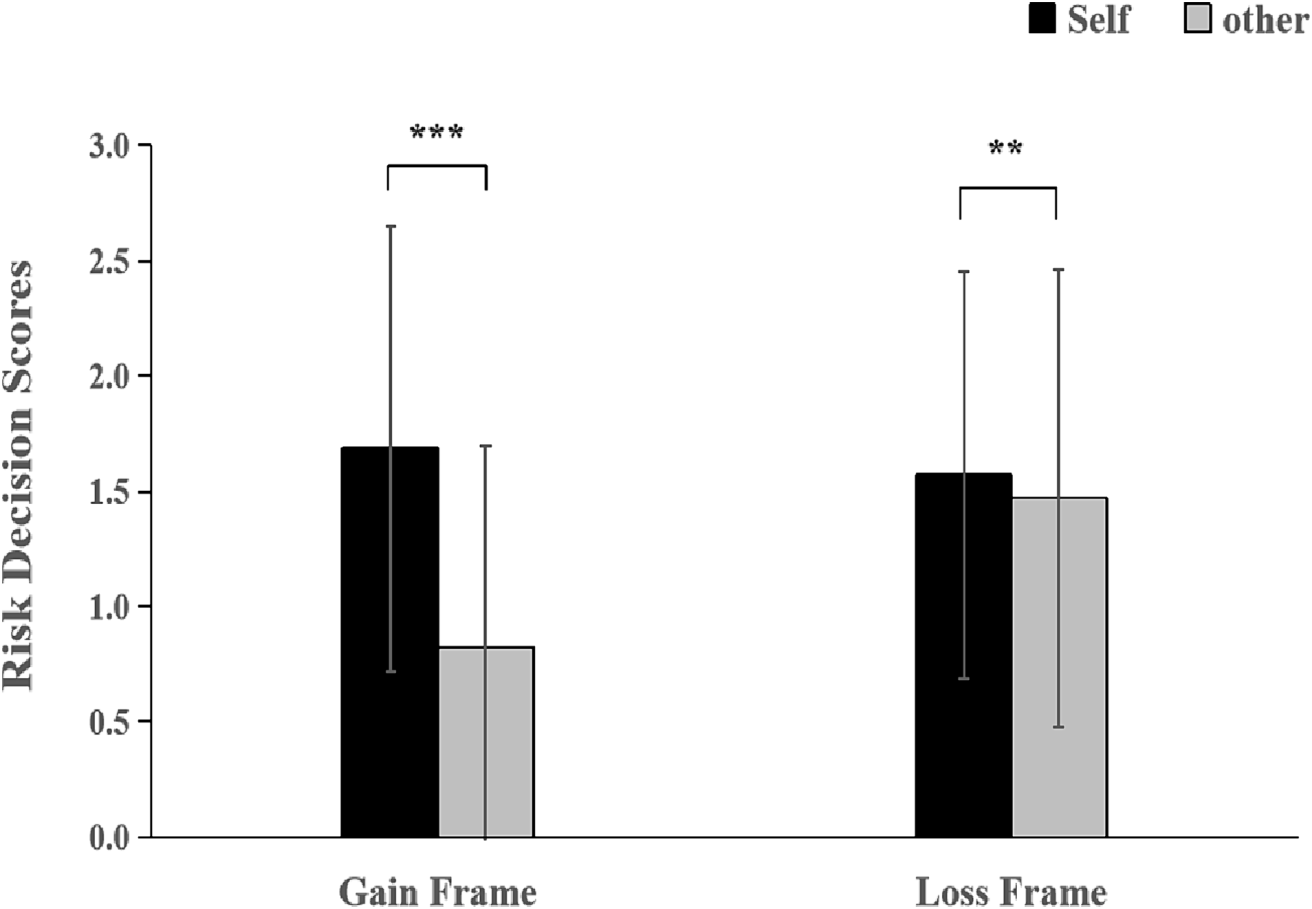
Self-other risk decision scores differ under various decision frame. Note: **indicates *p* < 0.01, ***indicates *p* < 0.001.

The results of the repeated measures analysis of variance (ANOVA) for the 2 (Frame Condition: Gain, Loss) × 2 (Decision Target: Self, Other) under the experiential information presentation revealed that the main effect of Frame Condition was not significant, *F*(1, 67) = 3.50, *p* = 0.065. However, the main effect of Decision Target was significant, *F*(1, 67) = 7.78, *p* = 0.007. Regardless of the context, participants showed higher risk scores when making decisions for themselves (*M* = 1.63, *SD* = 0.95) compared to decisions for others (*M* = 1.15, *SD* = 0.90). The interaction effect between Decision Target and Frame Condition was significant, *F*(1, 67) = 7.18, *p* = 0.009, *η_p_^2^* = 0.01. Further simple effects analysis revealed that in the gain condition, participants had higher risk scores when deciding for themselves (*M* = 1.69, *SD* = 0.96) compared to deciding for others (*M* = 0.82, *SD* = 0.87). When deciding for others, in the loss condition, risk scores (*M* = 1.47, *SD* = 0.99) were higher than in the gain condition (*M* = 0.82, *SD* = 0.87) (Figure 2).

### 2.4 Discussion

Experiment 1 investigated the impact of experiential information presentation on risk decision-making for oneself and others. The results revealed individual differences in decision-making for oneself and others, particularly in gain situations. Compared to decisions for oneself, participants exhibited more risk aversion when making decisions for others, a finding consistent with Olschewski et al.’s (2019) research. Although Experiment 1 identified self-other decision differences in experiential information, real-life decision-making often involves a mixture of various forms of information, rather than just descriptive or experiential information alone. Therefore, Experiment 2, building on these findings, further explored how simultaneous presentation of descriptive and experiential information would influence participants’ risk decision preferences for oneself and others. The study aimed to investigate which information presentation format had a greater impact on decision-making preferences in real-world scenarios.

## 3 Experiment 2: the simultaneous presentation of two types of information and its impact on self-other risk decision-making

### 3.1 Purpose and hypothesis

Adopting a 2 (Decision Target: Self vs. Other) × 2 (Frame Condition: Gain vs. Loss) mixed-design experiment. The independent variables were Decision Target (between-group variable) and Frame Condition (within-group variable), with the dependent variable being risk preference. Adopting the simultaneous presentation of descriptive and experiential information, participants were tasked with making risk decisions for themselves and others. The study aimed to explore the impact of these two information presentation methods on self-other risk decision-making. It was hypothesized that when presenting two types of information simultaneously, there would be differences in risk preferences for oneself and others.

### 3.2 Methods

#### 3.2.1 Participants

Using G-power 3.1 software, the required total number of participants under the conditions of effect size (Effect size) of 0.25 and statistical power (Power) of 0.8 is 46. Eighty university students were recruited for the current experiment, including 39 females and 41 males, *M_age_* = 21.55, *SD* = 2.21. All participants were right-handed, and their vision or corrected vision was normal. Participants were randomly assigned to the self (*n* = 40) and other (*n* = 40) groups. All participants volunteered and had not participated in similar experiments before. The experiment obtained approval from the Ethics Committee of Northwest Normal University, and all participants provided written informed consent. Before the experiment, participants were informed that they would receive a compensation of 20 yuan upon completion of the experiment.

#### 3.2.2 Materials

The decision scenarios utilized classic monetary decision tasks as the research material. The premise framework for risk decision-making was divided into gain and loss, with each of these frames further categorized into conservative and risky options. The selection involved a binary choice, requiring participants to make decisions under different frameworks.

#### 3.2.3 Procedure

The experiment utilized the same risk decision-making scenario materials as Experiment 1, with the preparation section preceding the formal experiment being identical to that of Experiment 1. The experiment was conducted using E-Prime 2.0, starting with the presentation of instructions to the participants, followed by the provision of experiential information, and culminating in the formal decision-making process. The instructions alerted participants whether they were making decisions for themselves or for others. Following the instructions, descriptive information about the decision options was presented, providing statistical information about outcomes and probabilities derived from extensive prior experiments in a textual format. Participants were informed that this information was for reference only and did not represent real probabilities. To gain a more comprehensive understanding of the options, participants could later engage in a sampling phase by pressing corresponding buttons to view outcomes; after reading the descriptive information, participants entered the sampling phase, which was presented in the same manner as in Experiment 1, continuing until they felt they had a sufficient understanding of each option before moving on to the formal decision-making stage to make their selections based on this understanding.

For example, in decision problem 1, the descriptive information displayed on the screen might be: option F has an 80% probability of earning 25 yuan and a 20% probability of earning 0 yuan; option J has a 100% probability of earning 20 yuan. After reading, participants enter the sampling phase, where “F” and “J” buttons are displayed on the screen. Participants are required to press the corresponding button to make a sampling choice. After selecting an option, the system provides outcome feedback based on the preset probabilities. There is no time or number limit on the entire sampling process, until the participant feels that they have adequately understood each option. They then proceed to the formal choice phase, choosing between Options F and J without feedback.

### 3.3 Results

In the analysis where both experiential and descriptive information were presented simultaneously, a repeated-measures analysis of variance for 2 (Frame Condition: Gain, Loss) × 2 (Decision Target: Self, Other) was performed. Analysis determined that none of the main or interaction effects reached significance: Frame Condition, *F*(1, 78) = 2.39, *p* = 0.13; Decision Target, *F*(1, 78) = 0.02, *p* = 0.88; and the interaction between Frame Condition and Decision Target, *F*(1, 78) = 1.06, *p* = 0.31.

**Figure 3:**
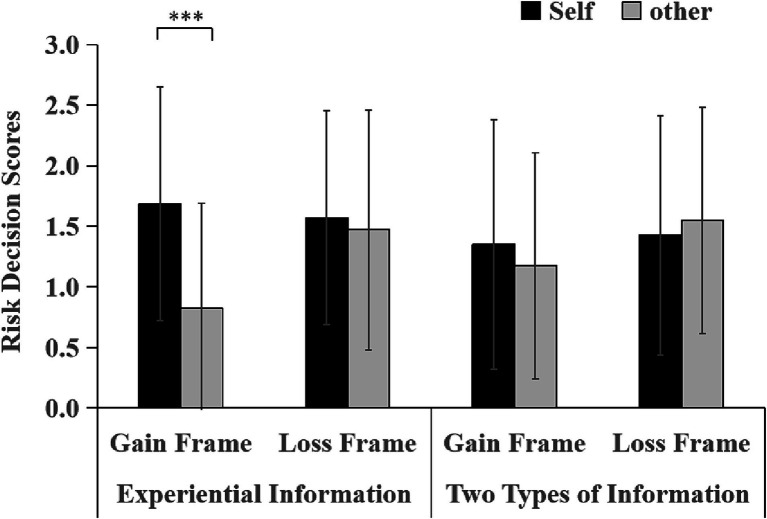
Self-other risk decision scores in different decision frames with varying information presentation. Note: ***indicates *p* < 0.001.

Furthermore, comparing the results of Experiment 1 with Experiment 2, a repeated-measures analysis of variance was conducted for 2 (Frame Condition: Gain, Loss) × 2 (Decision Target: Self, Other) × 2 (Information Presentation: Experiential Information, Two Types of Information). The findings were as follows: the main effect of Frame Condition reached statistical significance, *F*(1, 145) = 5.75, *p* = 0.02, *η_p_^2^* = 0.04, indicating a notable difference in the effect of gain versus loss frames on decision-making. The main effect of Decision Target was also significant, *F*(1, 145) = 4.61, *p* = 0.03, *η_p_^2^* = 0.03, highlighting differences in decision-making when the target is the self versus another person. The main effect of Information Presentation did not achieve significance, *F*(1, 145) = 0.01, *p* = 0.91, suggesting that the type of information presented (experiential versus both) did not independently affect decision-making outcomes. No significant interaction was observed between Frame Condition and Information Presentation, *F*(1, 145) = 0.04, *p* = 0.84, nor between Decision Target and Information Presentation, *F*(1, 145) = 3.75, *p* = 0.06. A significant interaction was found between Frame Condition and Decision Target, *F*(1, 145) = 6.71, *p* = 0.01, *η_p_^2^* = 0.04, indicating that the influence of the frame condition on decision-making varies depending on whether decisions are made for oneself or for others. Further analysis on the simple effects within the gain condition revealed a significant discrepancy in risk preferences: the risk associated with decisions made for oneself (*M* = 1.52, *SD* = 0.11) was significantly greater than that for decisions made on behalf of others (*M* = 1.00, *SD* = 0.11). The investigation into the three-way interaction among Frame Condition, Information Presentation, and Decision Target did not yield significant results, *F*(1, 145) = 1.27, *p* = 0.26, indicating that the combined influence of these factors on decision-making did not significantly differ across conditions (Figure 3).

### 3.4 Discussion

The results of Experiment 2 revealed that participants’ risk preferences, after receiving both descriptive and experiential information, were similar to those who only received experiential information. This is noteworthy because existing research suggests differences in risk decision-making under descriptive and experiential information. However, in the current study, when both types of information were presented, participants’ behavior did not differ from those who only received experiential information. This finding suggests that, in the presence of various decision information presentations, individuals tend to rely more on experiential information. The experiment also found that in gain situations, participants exhibited a higher risk preference when making decisions for themselves, consistent with the results of Experiment 1.

## 4 General discussion

In prior research, there has been inconsistency in the results regarding decision-making for others. Some studies have found that participants exhibit greater risk preference when making decisions for others (Beisswanger et al., 2003; Wray and Stone, 2005; Stone et al., 2006), while others have found a reduced risk preference when deciding for others (Zaleska and Kogan, 1971; Petrova et al., 2016). According to the results of this study, psychological distance and information presentation are identified as contributing factors to these discrepancies. Experiment 1, through the manipulation of social distance between others and participants, explored varying risk preferences exhibited by participants for others with different social distances, revealing that participants tend to engage in decision behaviors for others who are socially closer to them. Experiment 2 further investigated whether this similar pattern would be influenced by the presentation of information, and the results indicated that experiential decision-making influenced a comparable pattern in participants’ decision-making for themselves and others.

Social distance represents the subjective closeness between oneself and a target individual (Nan, 2007). As social distance increases, predicting the intentions and thoughts of others becomes more challenging. Consequently, individuals may rely more on general social attitudes and norms (Ames, 2004; Mitchell et al., 2005; Kim and Schnall, 2021). The current study also found that participants exhibited more objective decision-making behaviors for strangers. Exploring the changes in decision preferences induced by this psychological distance can be approached from various perspectives. Firstly, from the standpoint of construal level theory, a component of psychological distance, it can be observed that psychological distance alters individuals’ focus from concrete and immediate issues to broader and more generalized goals and outcomes (Chen, 2020). Therefore, in risk tasks, an increase in social distance may lead participants, when deciding for others, to shift their attention from immediate gains or losses to considerations of justice or fairness. In Experiment 1, participants exhibited no differences in risk preferences for socially distant strangers in different frames, aligning with the assertions of construal level theory. Moreover, as social distance increases, individuals may adopt a different psychological perspective when making decisions for the target individual. When deciding for oneself or close others, decision-makers consider their own desires, while decisions for psychologically distant others involve considering the possible intentions from their own perspective, taking into account objective factors such as the decision context (Kruger and Gilovich, 2004). According to regulatory focus theory, individuals exhibit inconsistent risk preferences when deciding for themselves and others due to different decision motivations (Higgins, 1997). Decision processes involve defensive and promotive regulatory strategies. When deciding for oneself, individuals typically adopt a defensive strategy, fearing potential losses, leading to greater attention to negative outcomes. Notably, in the gain frame, participants displayed a more conservative risk preference when deciding for themselves and distant others, ensuring gains. In the loss frame, the increased risk preference aimed at potential gains. This suggests that participants employed similar strategies when deciding for themselves and socially distant others. The results of Experiment 2 indicated that participants exhibited similar behavioral patterns and potential decision motivations when deciding for themselves and others. However, this pattern was influenced by the presentation content, with experiential information altering this similarity in patterns.

In the context of risk decision-making, different ways of presenting information may lead to varying decisions. The typical presentation formats for decision information include descriptive information and experiential information. The distinction lies in descriptive information presenting authentic information for each choice in the decision task, while experiential decision-making does not provide complete information; instead, decision-makers need to acquire it through an experiential learning process (Hadar and Fox, 2009). Early studies using the descriptive information presentation format found that individuals tend to exhibit more risk preference when deciding for others (Chakravarty et al., 2011; Batteux et al., 2017), aligning with the Risk As Feelings theory. In Experiment 1, participants, after receiving experiential information, demonstrated more risk aversion when deciding for others in the gain frame. This differs from the performance of participants deciding for others in previous descriptive information conditions and in Experiment 2. One possible reason for this difference is that people often underestimate small probability events in experience-based decision tasks (Hertwig et al., 2004). In descriptive-based decision tasks, due to the certainty of probabilities, individuals are more inclined to believe in the occurrence of small probability events. However, in experience-based decision tasks, the opposite tends to happen. Research has found that in experiential decision scenarios, individuals tend to prefer riskier options when deciding for themselves. This preference for high-risk, high-return options may arise from the perception that the likelihood of additional events occurring is minimal in experiential tasks. In contrast, individuals tend to make stable choices in descriptive information contexts (Hertwig et al., 2004). Since the study involved decisions for socially distant others, participants might consider additional factors when making decisions for others in experiential tasks. While they may overlook the probability of risk when deciding for themselves, making decisions for unfamiliar others may evoke a greater sense of responsibility. The psychological guilt associated with making incorrect decisions for others can outweigh the satisfaction from making correct decisions (Baumeister et al., 2001). Additionally, participants might take into account the potential impact on the intimacy of their relationship with the unfamiliar others if their decisions were to adversely affect their interests. People living in collectivist cultures are less inclined to engage in actions that could jeopardize their relationships. They would opt for more cautious approaches to prevent potential negative outcomes, allowing them to provide reasonable justifications even if risks materialize. Moreover, experiential information enhances individuals’ perception of the existence of small probabilities and makes these probabilities more deeply ingrained in memory. This leads them to use these small probability events as prototypes when making decisions, resulting in similar choices, such as selecting riskier options in gain conditions and safer options in loss conditions.

When further employing decision scenarios that are more likely to occur in real life, i.e., presenting multiple types of decision information simultaneously, participants’ risk preferences did not differ when deciding for themselves or others compared to the decision behavior when only experiential information was presented. This suggests that descriptive information may not have influenced participants’ decision-making. In situations where multiple types of information are presented simultaneously, participants might rely more on experiential information. This aligns with the findings of Lejarraga et al. (2015), who investigated the descriptive-experiential gap in medical decision tasks and found that participants relied more on experiential information when making decisions. This seems to indicate that when making decisions, participants place a higher reliance on experiential information they have personally encountered than on provided probability information. The study also found that after presenting both types of information simultaneously, the self-other decision differences under experiential information conditions disappeared. This suggests that descriptive information influenced the self-other risk decision differences in the context of presenting multiple types of information. Possible reasons for this include the fact that descriptive information counteracted estimates of smaller probabilities in experiential information, pulling participants’ thinking back to reality. Experiential information reminded participants of small probability events they had experienced, and because memories of significant victories and losses are more profound, participants tend to make riskier choices to ensure greater gains or smaller losses. However, descriptive information prompts participants to consider potential future events more, and for the sake of result stability, they typically base their decisions on more certain references provided by the information (Ludvig and Spetch, 2011). Therefore, when both types of information are presented, participants’ thinking based on experiential information is constrained by the more realistic descriptive information.

This study also has some limitations, first, the role of others in this study was played by strangers, yet previous research has indicated that the essence of self-other decision-making differences lies in the psychological distance, which varies between different others and the subjects. Therefore, future studies could employ others with varying psychological distances, such as friends or close family members, to investigate the differences in decision-making between self and others. Second, in the decision-making tasks, this study only selected tasks within the monetary domain. However, in real-life situations, individuals’ decision-making domains are not limited to financial matters alone. For instance, decisions regarding social relationships, personal safety, and others require individuals to make choices for themselves or for others. Therefore, future research could expand on the types of decision-making tasks to explore differences in self-other decision-making across various domains. Third, this study merely explored the differences in self-other decision-making across different types of information at a phenomenological level. However, the underlying differences involve various cognitive processing modes and other factors. Future studies could delve into cognitive processing and other aspects to uncover the reasons behind these differences, thereby offering a more comprehensive theoretical explanation for the self-other decision-making discrepancies.

Moreover, people tend to make decisions based on past experiences and current information. When making decisions for others, considerations also extend to the intimacy of the relationship with the other person. China, being a collectivist culture, differs in the dynamics of self-other relationships compared to Western cultures. Therefore, there may be variations in performance when making risk decisions for others. Future research could incorporate cultural factors into the study of the relationship between social distance and risk decision-making to better analyze self-other risk decision behaviors. Additionally, when making decisions for others, individuals need to adopt a perspective that takes into account the other person’s point of view. Hence, the individual’s empathic abilities may influence decision-making behaviors. If an individual lacks strong empathic abilities, their decisions may differ from those with higher empathic capabilities. Future research in the field of self-other relationships could explore empathy as a contributing factor in decision-making processes.

## 5 Conclusion

After receiving experiential information, individuals exhibited greater risk aversion when making decisions for others in gain scenarios. When both types of information were presented simultaneously, individuals tended to rely more on experiential information to make decisions.

## Data availability statement

The datasets presented in this study can be found in online repositories https://figshare.com/s/9698c8accd3047243284.

## Ethics statement

The study protocol was approved by the Ethics Board of Northwest Normal University (ERB No. 2021076, dated on 06/07/2021). The studies were conducted in accordance with the local legislation and institutional requirements. The participants provided their written informed consent to participate in this study.

## Author contributions

A-BZ: Conceptualization, Data curation, Formal analysis, Funding acquisition, Writing – original draft, Writing – review & editing. Z-KL: Conceptualization, Investigation, Methodology, Project administration, Software, Writing – review & editing. PX: Conceptualization, Data curation, Formal analysis, Investigation, Writing – review & editing. Y-FL: Investigation, Methodology, Project administration, Writing – review & editing. B-XC: Investigation, Methodology, Project administration, Writing – review & editing. LY: Formal analysis, Project administration, Validation, Writing – review & editing. C-ZH: Conceptualization, Formal analysis, Resources, Supervision, Validation, Writing – original draft, Writing – review & editing.
